# Beyond uncertainty in modern active learning for trustworthy AI

**DOI:** 10.3389/frai.2026.1844765

**Published:** 2026-06-19

**Authors:** Ridha Horchani

**Affiliations:** Department of Physics, College of Science, Sultan Qaboos University, Muscat, Oman

**Keywords:** active learning, annotation cost, data-efficient learning, evaluation realism, human-in-the-loop machine learning, supervision allocation, trustworthy AI

## Abstract

Active learning (AL) is a central response to the annotation bottleneck in modern artificial intelligence: when labels are expensive, a learner should query for the most useful forms of supervision rather than indiscriminately acquiring labels. However, contemporary AL is no longer a unified field organized around a small set of stable query principles. It is fragmented across acquisition strategies, supervision granularities, operational regimes, and evaluation protocols, making reported gains difficult to compare and, in some cases, to trust. This study offers a critical review and synthesis of modern AL, with particular attention to deep learning and deployment-oriented applications across medical imaging, computer vision, natural language processing, systematic review automation, recommender systems, anomaly detection, and structured prediction. The review makes three contributions. First, it proposes a four-axis taxonomy organized around acquisition logic, supervision granularity, operational regime, and evaluation realism. Second, it compares major acquisition families, including uncertainty-based, disagreement-based, expected-improvement, representativeness-based, diversity-aware, cost-aware, and shift-aware approaches, highlighting their assumptions, strengths, computational trade-offs, and recurrent failure modes. Third, it distills design principles and an actionable research agenda for trustworthy AL, emphasizing annotation cost, redundancy control, robustness under distribution shift, fairness, human oversight, and workflow-grounded evaluation. The central argument is that the main challenge for AL has shifted from identifying informative samples to designing supervision-allocation pipelines whose gains remain reliable across realistic annotation workflows, heterogeneous human effort, and deployment constraints.

## 1 Introduction

The extraordinary performance of modern artificial intelligence (AI) systems often rests on a less visible but essential foundation: the availability of large, high-quality labeled datasets. Across domains such as medical imaging, industrial inspection, recommender systems, and document intelligence, this dependence on supervision remains a central bottleneck. Labels are not merely scarce in a statistical sense; they are often costly because they require time, money, domain expertise, adjudication, and repeated curation (Settles, 2009; Lewis and Gale, 1994; Seung et al., 1992; Cohn et al., 1996). In many applications, the central question is therefore not only how to train a better model but also how to allocate limited human labeling effort more rationally.

Active learning (AL) emerged at the intersection of statistical efficiency and annotation scarcity. Its classical intuition is simple: if some unlabeled examples are more informative to label than others, a learner should query those examples selectively rather than sample labels at random (Settles, 2009). From that starting point, AL has evolved into a broad and methodologically diverse field that includes uncertainty sampling, query-by-committee, expected error reduction, expected model change, Bayesian querying, core-set and diversity-based batch selection, adversarial and representation-based acquisition, cost-aware strategies, and shift-aware sampling (Roy and McCallum, 2001; Settles and Craven, 2008; Gal et al., 2017; Beluch et al., 2018; Sener and Savarese, 2018; Yoo and Kweon, 2019; Sinha et al., 2019; Ash et al., 2019).

This expansion has brought considerable innovation, but it has also made the field more difficult to interpret. Modern AL is no longer a compact methodological area organized around a small set of shared assumptions. It is now distributed across application domains, model families, annotation interfaces, supervision granularities, and evaluation protocols. A method designed for instance-level image classification may be discussed alongside methods for voxel-level segmentation, region-level object detection, token-level named entity recognition, or ranking-based medical assessment, even though the operational meaning and cost of a single query differ substantially across these settings. Moreover, recent work has shown that reported AL gains can be highly sensitive to evaluation assumptions, including random seeds, budget schedules, batch composition, annotation cost, class imbalance, and distribution shift (Wang et al., 2024; Wu et al., 2022; Garcia et al., 2023; Mittal et al., 2025; Gentile et al., 2024).

This study is presented as a structured critical review rather than as benchmark research or a primary algorithmic contribution. Its purpose is not to introduce a new AL method, but to provide a conceptually integrated and practically grounded synthesis of the modern field. This study makes three contributions. First, it develops a taxonomy of modern AL along four axes: acquisition logic, supervision granularity, operational regime, and evaluation realism. Second, it offers a comparative analysis of the major method families, with attention to their assumptions, strengths, computational trade-offs, and recurrent failure modes. Third, it synthesizes the literature into design principles and open problems for trustworthy, human-centered, and deployment-relevant AL.

The contribution of this review is therefore integrative and analytical rather than algorithmic. Several prior surveys have reviewed classical active-learning principles and query strategies (Settles, 2009), deep active-learning architectures and acquisition functions (Wu et al., 2022; Wang et al., 2024), and application-specific developments, particularly in computer vision, object detection, and medical image analysis (Garcia et al., 2023). The present article complements those surveys by treating AL primarily as a problem of trustworthy supervision allocation rather than as a catalog of query heuristics.

In this review, Trustworthy AI refers to the design and evaluation of AI systems whose behavior remains reliable, robust, transparent, fair, safe, and accountable under realistic use conditions. These concepts are directly relevant to AL because query policies determine which parts of the data distribution receive human attention, expert labeling, verification, or correction. Reliability concerns the stability of reported label-efficiency gains across datasets, seeds, models, and budgets; robustness concerns sensitivity to noise, imbalance, calibration error, redundancy, and distribution shift; fairness concerns the representation of rare or high-risk sub-populations; transparency concerns the explainability of query decisions; human oversight concerns the role of annotators and adjudicators; safety concerns the risk of under-querying important cases; and accountability concerns whether annotation assumptions, query policies, and evaluation protocols are documented for scrutiny.

Against this background, the value of an AL method depends not only on how samples are selected but also on the form of human effort required, the operational conditions under which querying occurs, and the credibility with which the reported gains are evaluated. Table 1 summarizes this distinction relative to prior survey traditions.

**Table 1:** Positioning of the present review relative to prior active-learning surveys.

Review type	Typical emphasis	Emphasis of the present review
Classical active-learning surveys	Formal learning scenarios, foundational query strategies, and pool-based or stream-based selective sampling.	Uses these foundations as background, but focuses on how AL changes under deep-learning, workflow, and deployment constraints.
Deep active-learning surveys	Neural-network uncertainty, Bayesian approximation, ensemble methods, representation learning, core-set methods, and benchmark performance.	Treats deep acquisition methods as one component of a broader supervision-allocation pipeline involving cost, redundancy, robustness, and evaluation realism.
Application-specific surveys	Domain-specific methods in medical imaging, computer vision, natural language processing, or related areas.	Compares domains through a common analytical lens based on query unit, annotation burden, workflow constraints, and trustworthiness requirements.
Practical-challenge surveys	Label cost, data imbalance, model uncertainty, annotation noise, and deployment limitations.	Reorganizes these issues into a four-axis taxonomy and connects them explicitly to reliability, robustness, fairness, human oversight, safety, and accountability.

The central claim of the review follows from this comparison: supervision cost, robustness, fairness, and deployment realism should not be treated as peripheral implementation details but as organizing dimensions of AL itself. The proposed four-axis framework is therefore diagnostic. It asks not only which acquisition score a method uses but also what supervision it requests, which operating regime it assumes, and how realistic the supporting evidence is.

**Figure 1:**
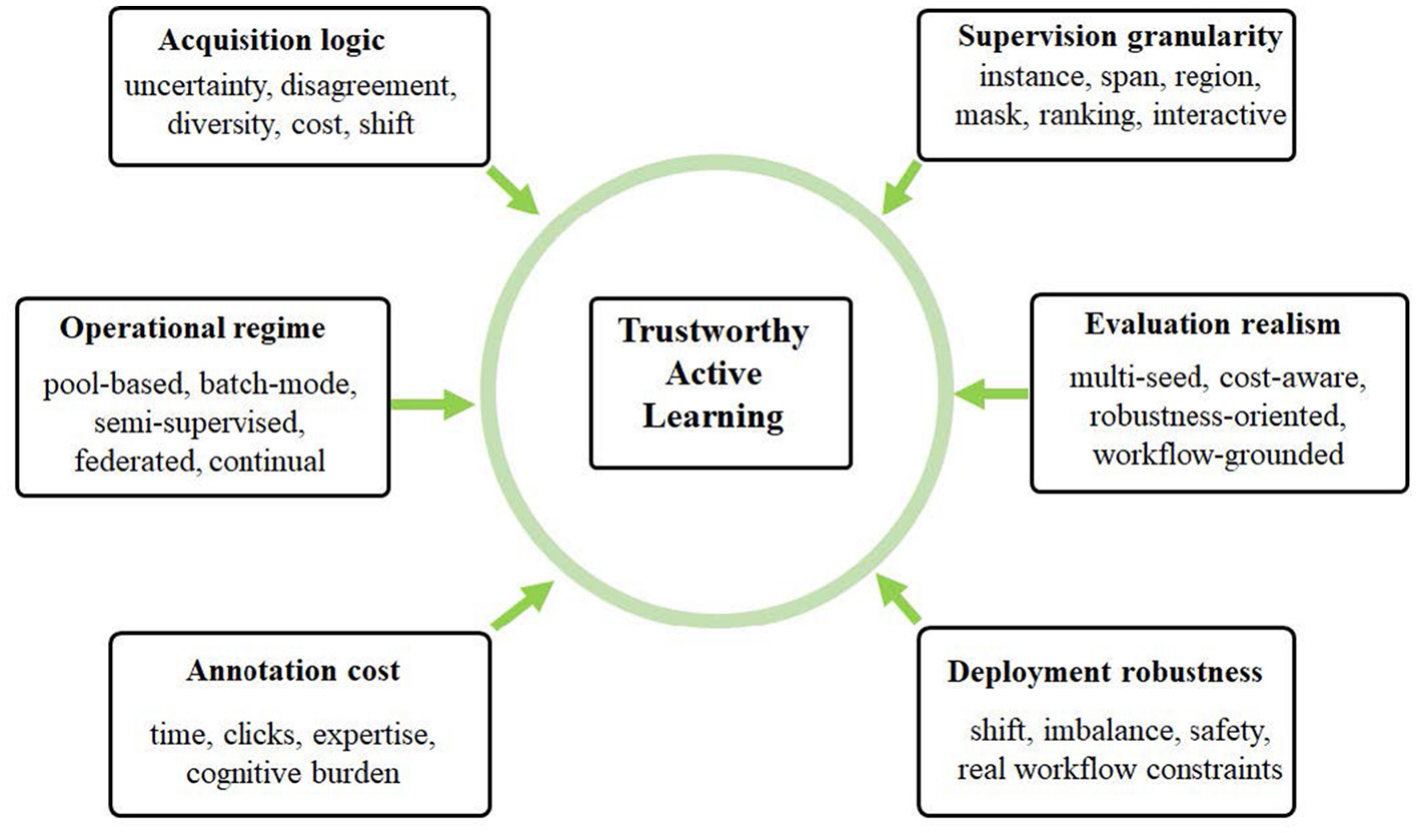
Conceptual framework for trustworthy AL. The figure summarizes the central claim of this review: AL is best understood not as a one-dimensional uncertainty-driven procedure but as a supervision-allocation framework shaped jointly by acquisition logic, supervision granularity, operational regime, evaluation realism, annotation cost, and deployment robustness.

To clarify this argument visually, Figure 1 presents the conceptual framework for trustworthy AL used in this review. The figure emphasizes that practical AL should be organized not around uncertainty alone, but around the interaction among acquisition logic, supervision granularity, operational regime, evaluation realism, annotation cost, and deployment robustness.

The remainder of the study is organized as follows. Section 2 presents the four-axis taxonomy. Section 3 develops a critical comparative analysis of the major acquisition families. Section 4 reviews domain-specific evidence across major AI application areas. Section 5 analyzes evaluation realism and methodological fragility in the literature. Section 6 distills design principles for trustworthy AL. Section 7 outlines a research agenda, and Section 8 concludes.

## 2 A systematic four-axis taxonomy of modern active learning

This review organizes the modern AL literature along four complementary axes: acquisition logic, supervision granularity, operational regime, and evaluation realism. These axes do not replace existing classifications by query strategy or application domain; rather, they extend them by asking how a query is selected, what form of supervision it purchases, where the learning process takes place, and how credible the reported gain is. The four axes proposed here are not intended as an exhaustive list of every factor that can influence AL. Rather, they identify four irreducible questions that any AL system must answer. Acquisition logic asks: Why should this candidate be queried? Supervision granularity asks: What exactly is being requested from the human oracle? Operational regime asks: Under what learning and deployment conditions is querying performed? Evaluation realism asks: How credible is the claimed gain? These questions are necessary because an AL method cannot be interpreted from its query score; its meaning also depends on the label being purchased, the workflow in which the query is embedded, and the evidential standard used to assess success.

Other dimensions, including model family, annotation interface, human expertise level, domain risk, and foundation-model adaptation, are important but are treated here as cross-cutting modifiers rather than primary axes. Model family primarily affects acquisition logic and uncertainty calibration; annotation interface and expertise affect supervision granularity and cost; domain risk affects evaluation realism and acceptable stopping criteria; and foundation-model adaptation modifies both acquisition logic and operational regime. The four-axis framework is therefore intended as a compact analytical structure that can absorb these additional dimensions without reducing AL to any single one of them. The categories below should also be read as analytical dimensions rather than mutually exclusive boxes, because practical AL systems often combine uncertainty with diversity, cost-aware selection with batch-mode querying, or semi-supervised learning with continual adaptation. Table 2 summarizes the proposed four-axis taxonomy and clarifies the role of each axis in interpreting modern active learning systems.

**Table 2:** Overview of a four-axis taxonomy for modern active learning.

Axis	Categories	What the axis clarifies
Acquisition logic	Uncertainty, disagreement, expected-improvement, representativeness, diversity-aware, cost-aware, shift-aware,	Why a query is selected and what notion of learning value it presupposes.
Supervision granularity	Instance, token/span, region/proposal, pixel/voxel/superpixel, pairwise/ranking, weak/interactive	What type of supervision is being purchased and how burdensome it is likely to be.
Operational regime	Pool-based, stream-based, batch-mode, semi-supervised, federated/privacy-sensitive, continual/shift-aware	under what learning and deployment setting the method operates.
Evaluation realism	Idealized i.i.d., multi-seed/multi-budget, cost-aware, robustness-oriented, workflow-grounded	how strong and transferable the reported evidence really is.

### 2.1 Acquisition logic

The first axis concerns why an unlabeled sample is selected, that is, what notion of learning value the acquisition rule presupposes. Table 3 compares the major acquisition families that structure the modern AL literature.

**Table 3:** Comparison of acquisition families in modern active learning.

Category	Description	Main strength	Main limitation
Uncertainty-based	Select samples for which the current model is least certain, using scores such as least confidence, predictive entropy, margin uncertainty, dropout variance, or mutual information (Lewis and Gale, 1994; Gal et al., 2017).	Simple, portable, and easy to integrate into standard pipelines.	Uncertainty is only a proxy for value and may be noisy, miscalibrated, or distorted by outliers in deep settings.
Disagreement-based	Prioritize points on which multiple predictors disagree, whether through committees, ensembles, or stochastic forward passes (Seung et al., 1992; Beluch et al., 2018).	Can capture epistemic uncertainty better than a single posterior confidence score.	Computationally more expensive and still sensitive to model misspecification.
Expected-improvement	Use criteria such as expected error reduction or expected model change to align acquisition directly with learning progress (Roy and McCallum, 2001; Settles and Craven, 2008).	Conceptually direct and closely tied to the intended objective.	Often expensive or unstable to estimate in deep settings.
Representativeness-based	Favor samples that are central, typical, or structurally important within the unlabeled distribution (Sener and Savarese, 2018).	Can improve coverage of the data distribution.	Typicality does not guarantee utility.
Diversity-aware	Constrain batch composition so that queried points are not redundant; includes core-set, gradient-embedding, clustering-based, and submodular approaches (Sener and Savarese, 2018; Ash et al., 2019; Gentile et al., 2024).	Especially valuable in realistic batch annotation workflows.	Diversity alone does not ensure informativeness.
Cost-aware	Incorporate heterogeneous labeling cost explicitly, reframing acquisition as expected gain per unit of effort (Wang and Stavness, 2025; Arefeen and Ghasemzadeh, 2025).	Aligns the method more closely with real annotation constraints.	Requires credible and often task-specific cost modeling.
Shift-aware/rarity-aware	Adapt acquisition to class imbalance, rare-event detection, or mismatch between the unlabeled pool and the deployment distribution (Yang et al., 2025).	Better suited to non-i.i.d. and deployment-relevant settings.	Often depends on additional assumptions and less standardized evaluation.

The strengths and limitations in Table 3 are therefore indicative rather than absolute. In practice, the behavior of each family depends on dataset geometry, model calibration, architecture, labeling noise, class imbalance, batch size, and the annotation interface. The table is intended to clarify dominant tendencies, not to rank methods universally.

### 2.2 Supervision granularity

The second axis concerns what kind of supervision is being purchased, and therefore what sort of human effort a “query” entails. Methods that appear comparable at the level of query count may, in fact, require very different forms and amounts of annotation work. Table 4 summarizes the main supervision granularities encountered in modern AL.

**Table 4:** Comparison of supervision granularities in modern active learning.

Category	Description	Typical applications	Main implication
Instance-level labels	A query returns a single label for an entire image, document, or example.	Standard classification tasks.	Usually the simplest and cheapest form of supervision, though still potentially expertise-dependent.
Token- or span-level labels	Annotation targets spans, entities, or sequence fragments rather than full documents (Riesener et al., 2024).	Natural language processing and document intelligence.	More labor-intensive than document-level labels because the annotator must localize and mark internal structure.
Region- or proposal-level labels	The queried object is a region proposal or local object annotation rather than the entire image (Chen et al., 2023).	Object detection and weak localization.	Requires spatial localization, making annotation more costly than simple instance labeling.
Pixel-, voxel-, patch-, or superpixel-level labels	Supervision is dense and may involve full masks, slices, patches, or superpixels (Gaillochet et al., 2023; Li et al., 2023; Wang and Stavness, 2025).	Semantic and medical image segmentation.	Often among the most expensive forms of supervision because it demands fine-grained spatial delineation.
Pairwise or ranking labels	The oracle provides relative judgments rather than absolute class labels (Kadota et al., 2024).	Ranking-based medical assessment and preference-sensitive tasks.	Effort depends on the complexity of the comparison and may be cognitively demanding even when the annotation is brief.
Weak or interactive labels	The oracle provides partial, corrective, or approximate labels rather than complete annotations (Li et al., 2023).	Interactive annotation and weak-supervision workflows.	Can substantially reduce effort per query, but may require more careful downstream aggregation or correction.

Annotation burden is not determined by granularity alone. It also depends on annotator expertise, interface design, fatigue, quality-control procedures, disagreement resolution, adjudication, and downstream correction effort. For this reason, cost-aware AL should ideally measure or model human effort directly rather than assume that all queries within a granularity class are equally expensive.

### 2.3 Operational regime

The third axis concerns the broader learning setting in which AL operates. Methods that appear similar at the level of acquisition scores may behave quite differently once embedded in distinct operational regimes. Table 5 summarizes the main regimes discussed in the literature.

**Table 5:** Comparison of operational regimes in modern active learning.

Category	Description	Typical setting	Main implication
Pool-based active learning	The learner repeatedly selects queries from a large static pool of unlabeled data (Settles, 2009).	Standard offline active learning benchmarks and many applied pipelines.	Conceptually simple and widely used, but may underrepresent non-stationary or streaming environments.
Stream-based active learning	Data are encountered sequentially, and the learner decides online whether the current sample should be labeled (Settles, 2009).	Online systems and temporally ordered data streams.	Better suited to real-time settings, but queying decisions must be made without access to the full unlabeled pool.
Batch-mode active learning	The learner queries sets of samples at once rather than one by one (Ash et al., 2019; Gentile et al., 2024).	Most practical human annotation workflows.	Reflects real operational constraints, but requires explicit control of redundancy and batch diversity.
Semi-supervised active learning	Querying is combined with pseudo-labeling, consistency regularization, or other uses of unlabeled data (Sinha et al., 2019; Chen et al., 2023).	Data-efficient learning settings with abundant unlabeled data.	Can improve label efficiency but makes attributing gains between querying and unlabeled-data exploitation more complex.
Federated or privacy-sensitive active learning	Querying occurs under distributed data access, local storage constraints, or privacy limitations (Deng et al., 2025).	Federated learning, clinical systems, and privacy-sensitive applications.	Better aligned with constrained data governance, but complicates acquisition design and coordination.
Continual or shift-aware active learning	The learner operates under evolving distributions, target-domain adaptation, or changing task priorities (Yang et al., 2025).	Non-stationary environments and deployment-sensitive systems.	More realistic for long-term use, but evaluation and benchmarking are less standardized.

These regimes should not be read as mutually exclusive. In practice, modern AL systems often combine batch-mode querying with semi-supervised learning, operate under privacy-sensitive constraints, or require continual adaptation after deployment. The operational-regime axis is therefore intended to identify the assumptions under which querying occurs, rather than to assign each method to a single, rigid category. This distinction is crucial because the same acquisition score may have different practical implications in an offline pool, a federated clinical system, or a non-stationary deployment environment.

### 2.4 Evaluation realism

The fourth axis addresses the credibility and transferability of the reported empirical evidence. Because many apparent gains in AL depend as much on evaluation design as on acquisition logic, this axis is essential for distinguishing robust findings from fragile benchmark results. Table 6 summarizes the main levels of evaluation realism in the literature.

**Table 6:** Comparison of evaluation modes in modern active learning.

Category	Description	What it captures	Main limitation
Idealized i.i.d. evaluation	Assumes a clean, unlabeled pool, homogeneous label cost, stable architectures, and favorable train–test alignment.	Useful for initial comparison under controlled conditions.	Often overstates robustness and practical utility in real-world settings.
Multi-seed and multi-budget evaluation	Reports performance across different random seeds, initial labeled sets, and budget levels (Mittal et al., 2025).	Reveals sensitivity and variance hidden by single-run performance curves.	Still may omit realistic annotation cost or deployment mismatch.
Cost-aware evaluation	Relates performance not only to the number of queries but also to actual or modeled annotation effort (Wang and Stavness, 2025; Arefeen and Ghasemzadeh, 2025; Bonković et al., 2026).	Better reflects the practical efficiency of active learning.	Depends on credible and often task-specific cost models.
Robustness-oriented evaluation	Includes class imbalance, label noise, calibration issues, distribution shift, or cross-model variation (Mittal et al., 2025; Yang et al., 2025).	Tests whether gains remain stable under more realistic perturbations.	Can be harder to standardize across datasets and application domains.
Workflow-grounded evaluation	Ties AL directly to a real human process, such as systematic review screening or interactive medical labeling (Ferdinands et al., 2023; Teijema et al., 2025).	Provides the most direct estimate of time savings, effort reduction, or operational value.	Usually domain-specific and less easily comparable across tasks.

The evaluation-realism axis is introduced here as part of the taxonomy, but its methodological implications are developed in greater detail in Section 5. Its inclusion at the taxonomy level reflects a central claim of this review: evaluation design is not a secondary reporting issue but part of how an AL method should be interpreted. A method supported only by single-seed, label-count-based, i.i.d. benchmarks provides weaker evidence of trustworthy supervision allocation than one evaluated under cost-aware, multi-budget, robustness-oriented, or workflow-grounded conditions.

Taken together, these four axes show that active learning methods cannot be adequately compared based on acquisition logic alone. A method's significance depends not only on how it scores candidate queries but also on the form of supervision it requires, the operational regime in which it is deployed, and the realism with which its gains are evaluated. This taxonomy, therefore, provides the analytical foundation for the comparative discussion, domain-specific synthesis, and evaluation critique developed in the following sections.

## 3 A critical comparative analysis of major acquisition families

The taxonomy developed in the previous section provides an organizing structure for modern AL, but classification alone is insufficient. A review must also ask when different acquisition families are likely to succeed, what assumptions they rely on, and whether their reported advantages remain meaningful under realistic annotation and deployment constraints. The central issue is therefore not only how an acquisition rule is defined but also what learning value it captures, what it ignores, and how fragile it may be to calibration error, redundancy, cost variation, class imbalance, or distribution shift.

This section compares the major acquisition families as partially complementary approaches to defining learning value. Rather than treating them as isolated algorithmic categories, the discussion emphasizes their practical trade-offs and their role in the broader shift from single-score sample ranking to multi-objective supervision allocation.

### 3.1 Uncertainty, disagreement, and expected improvement

Uncertainty-based AL remains the most widely used family because it is simple, intuitive, and easily integrated into standard predictive pipelines. In classification settings, the learner can query the highest-entropy examples, the smallest-margin examples, or the least confident predictions (Lewis and Gale, 1994; Gal et al., 2017). In many benchmark settings, these methods yield respectable label-efficiency gains and provide strong baselines against which more complex acquisition functions should be compared.

Their main weakness, however, is both conceptual and empirical. Uncertainty is not identical to expected utility. A point may be uncertain because it lies near a decision boundary, because it is genuinely informative, because it is mislabeled or corrupted, because it is out of distribution, or because the model is poorly calibrated. In deep networks, overconfidence, representation instability, and poor calibration can render raw uncertainty a fragile signal (Beluch et al., 2018; Mittal et al., 2025). Moreover, in batch settings, the most uncertain points may cluster in a narrow region of the feature space, so naive top-*k* uncertainty sampling can waste budget on redundant examples.

Disagreement-based methods extend the concept of uncertainty by comparing multiple plausible learners. Query-by-committee is the classic example (Seung et al., 1992); modern variants use ensembles, stochastic subnetworks, or repeated stochastic forward passes (Beluch et al., 2018). Their advantage is that disagreement can more directly approximate epistemic uncertainty than a single confidence score, especially in low-label regimes. Nevertheless, disagreement is not immune to the limitations of uncertainty. It can still reflect noise, distributional mismatch, poor representation, or insufficient committee diversity. Moreover, it is computationally more expensive than single-model uncertainty scoring, especially when multiple models must be trained or evaluated.

Expected-improvement methods seek a more direct notion of acquisition value. Criteria such as expected error reduction or expected model change estimate how much a candidate label would improve the learner (Roy and McCallum, 2001; Settles and Craven, 2008). In principle, these methods are attractive because they align the acquisition rule with the intended learning objective. In practice, however, estimating the downstream effect of a candidate label is computationally expensive, particularly in deep learning. The required approximations may be unstable, and the added complexity does not always yield more robust empirical gains. For this reason, expected-improvement methods remain conceptually important, but in many applied deep AL pipelines, they are replaced or approximated by more tractable hybrid heuristics.

Taken together, these three families illustrate a central lesson: informativeness is essential yet incomplete. Uncertainty, disagreement, and expected improvement all attempt to estimate how much a label may help the model, but none directly guarantees diversity, cost-effectiveness, robustness, or deployment relevance. Modern AL, therefore, increasingly combines these signals with additional constraints.

### 3.2 Representativeness and diversity in batch selection

Representativeness-based methods select samples that are central, typical, or structurally important within the unlabeled distribution (Sener and Savarese, 2018). Their motivation differs from that of uncertainty-based querying. Rather than asking where the model is least confident, they ask which samples best cover the data manifold. This is particularly useful in early acquisition rounds, when the labeled set is small, and the learner may not yet have a stable representation of the data distribution.

The limitation is that typicality does not imply utility. Once the model has captured the dominant structure of the data, additional representative points may offer limited learning benefit. Conversely, rare, ambiguous, or boundary cases may be highly informative precisely because they are not typical. Representativeness is therefore best understood as a corrective to uncertainty-only querying rather than as a sufficient acquisition principle in its own right.

Diversity-aware methods are especially important in batch-mode AL. Human annotation workflows rarely operate on a single point at a time, and retraining after each new label is often impractical. Batch-mode AL, therefore, requires the learner to select a set of examples that is not merely individually informative but collectively useful. Diversity-aware methods address this by clustering, core-set coverage, gradient-space embeddings, sub-modular objectives, or explicit redundancy penalties (Sener and Savarese, 2018; Ash et al., 2019; Gentile et al., 2024).

The importance of diversity is structural rather than incidental. A batch selected solely by uncertainty may contain many near-duplicate examples, whereas a batch selected solely by representativeness may avoid difficult or decision-critical cases. The strongest batch-oriented methods, therefore, treat diversity as a composition principle that must be balanced with informativeness. This is one reason modern AL has moved away from one-dimensional acquisition scores toward composite objectives that combine uncertainty, coverage, diversity, and task-specific utility.

### 3.3 Cost-aware, shift-aware, and rarity-aware acquisition

Cost-aware AL marks a deeper shift in the field's logic. It begins with the recognition that the unit of interest is not the label itself, but the human effort required to obtain it. A document label, a bounding box, a segmentation mask, a token span, and a medical ranking judgment should not be treated as equivalent simply because each counts as one query (Wang and Stavness, 2025; Arefeen and Ghasemzadeh, 2025; Bonković et al., 2026). Cost-aware acquisition instead reframes selection as a problem of expected gain per unit of annotation effort.

This shift is important because many practical AL claims depend on how cost is measured. A method that reduces the number of queried images may not reduce expert time if each selected image requires dense segmentation or difficult adjudication. Conversely, weak, partial, or interactive supervision may yield smaller per-query gains but greater efficiency per unit of effort. The methodological challenge is that cost is multidimensional. It may involve time, clicks, spatial complexity, cognitive load, fatigue, annotator expertise, disagreement resolution, and downstream correction. Cost-aware AL is therefore essential for deployment, but it is credible only when its cost model is explicit and realistic.

Shift-aware and rarity-aware methods make a parallel correction to the idealized i.i.d. assumptions underlying many AL benchmarks. In real applications, the unlabeled pool may not match the deployment distribution, and rare but important classes may be underrepresented. This is particularly important in medical diagnosis, industrial inspection, safety monitoring, rare-event detection, and domain adaptation. Shift-guided and rarity-aware acquisition methods attempt to prioritize target-relevant or underrepresented regions of the data space (Yang et al., 2025; Benhafid and Selouani, 2026).

Their promise is substantial, but their evaluation remains challenging. Unlike standard pool-based classification, shift-aware AL depends on assumptions about target relevance, rarity, or deployment risk. If these assumptions are poorly specified, an acquisition rule may appear useful in one benchmark but fail in another. For this reason, shift-aware and rarity-aware AL should be assessed not only by aggregate accuracy but also by rare-class coverage, robustness under distributional mismatch, and performance on operationally important subgroups.

### 3.4 Deployment-relevant trade-offs

A deployment-oriented comparison requires criteria that go beyond the acquisition score's name. Table 7 compares the main acquisition families across calibration dependence, redundancy sensitivity, computational burden, and deployment implications. The table is not intended as a quantitative meta-analysis; rather, it synthesizes recurring trends in the reviewed literature. It also clarifies the distinction between the taxonomy in Section 2 and the present comparative analysis: the taxonomy classifies AL systems, whereas this section evaluates their practical trade-offs.

**Table 7:** Analytical comparison of acquisition families according to deployment-relevant trade-offs.

Family	Calibration dependence	Redundancy sensitivity	Computational burden	Main deployment implication
Uncertainty-based	High: unreliable confidence can distort ranking	High in batch mode, because uncertain points may cluster	Low to moderate	Useful baseline, but should usually be paired with calibration checks and diversity control
Disagreement-based	Moderate to high: depends on committee diversity	Moderate, unless batch diversity is enforced	Moderate to high	Better proxy for epistemic uncertainty, but harder to deploy when inference or training cost is constrained
Expected-improvement	Objective-dependent rather than purely confidence-dependent	Moderate, depending on approximation	High	Conceptually strong, but often difficult to scale or estimate reliably in deep pipelines
Representativeness-based	Lower direct dependence on calibration	Lower for coverage, but may select weakly informative typical cases	Moderate	Useful for early exploration and redundant pools, but insufficient when rare or boundary cases matter
Diversity-aware	Indirect; depends on representation quality	Low when implemented properly	Moderate to high	Essential for batch annotation, but must be combined with informativeness or task utility
Cost-aware	Depends on the utility term used	Depends on the selected utility and cost model	Moderate	Most aligned with real annotation constraints, but only credible if effort is measured or realistically modeled
Shift-/rarity-aware	Depends on target-domain or rarity estimates	Variable	Moderate	Important for non-i.i.d. deployment and rare-event coverage, but requires explicit assumptions about target relevance

Analytical comparison of acquisition families according to deployment-relevant trade-offs.

### 3.5 Comparative synthesis

Several conclusions follow from this comparison. First, uncertainty-based querying remains useful but is rarely adequate as a standalone strategy in realistic settings. Its simplicity makes it a strong baseline, yet its dependence on calibration and sensitivity to redundancy limit its practical value. Second, disagreement-based and expected-improvement methods offer richer notions of informativeness, but they often increase computational complexity and still require safeguards against noise, misspecification, and shift. Third, representativeness and diversity are indispensable for coverage and batch construction, yet neither is sufficient without a task-relevant measure of informativeness. Fourth, cost-aware and shift-aware thinking should no longer be regarded as niche extensions; they increasingly define the practical future of AL because they address the human and deployment conditions under which supervision is actually produced.

A compact formal lens can summarize this composite trend. For a candidate query *x* from unlabeled pool *U* relative to labeled set *L*, one may write


$$\begin{array}{l} A\left(x;L,U\right)=\alpha U\left(x\right)+\beta R\left(x\right)+\gamma V\left(x;B,L\right)-\delta C\left(x\right)+\eta S\left(x\right), \end{array}$$ (1)

where U denotes informativeness or uncertainty, R representativeness, V diversity relative to the batch, C annotation cost, and S a shift-, rarity-, or robustness-related term. Equation 1 is not introduced here as a new algorithm. It is a synthetic expression for a field-wide methodological tendency: modern AL is increasingly a multi-objective supervision-allocation problem rather than a one-score ranking problem.

This synthesis also explains why evaluating AL methods must go beyond label efficiency alone. If acquisition is multi-objective, evidence of success should be multi-dimensional. A method should be assessed not only by accuracy per queried label but also by annotation effort, batch redundancy, calibration sensitivity, robustness under distribution shift, and suitability for the intended workflow. The following sections develop these implications across application domains and evaluation practices.

## 4 Domain-specific evidence across AI applications

The practical significance of AL becomes clearest when it is examined across application domains. Modern AL is no longer a narrow extension of classifier design; it is a family of supervision-allocation strategies used wherever labels are expensive, expertise-dependent, ambiguous, or operationally constrained. Simultaneously, domain evidence shows that AL does not transfer unchanged from one task to another. The meaning of a query depends on the annotation interface, the supervision unit, the data structure, the available expertise, and the evaluation criterion. For this reason, domain-specific evidence is not merely illustrative; it is central to understanding how AL functions in practice.

Because the meaning of a query varies substantially across application areas, Table 8 compares major domains in terms of supervision unit, annotation bottleneck, and evaluation criterion. The table provides the cross-domain basis for the supervision-allocation view developed throughout this review.

**Table 8:** Cross-domain analytical matrix for active learning as supervision allocation.

Domain	Typical supervision unit	Dominant bottleneck	Most informative evaluation criterion
Medical imaging	Mask, slice, voxel, patch, or superpixel	Expert time, spatial delineation, adjudication, and clinical risk	Performance per expert effort, robustness, and clinically meaningful segmentation or classification quality
Object detection	Image, region, bounding box, or proposal	Localization effort, object density, small objects, and class imbalance	Detection quality per annotation time or localization cost
NLP and document intelligence	Token, span, sentence, document, or relation	Semantic ambiguity, expertise, context dependence, and disagreement	Task performance per interpretive effort and robustness across corpora
Systematic review automation	Abstract, full-text record, or relevance judgment	Reviewer workload, prevalence, stopping rule, and recall requirement	Work saved at high recall and transparent stopping behavior
Structured prediction and anomaly detection	Match judgment, anomaly confirmation, ranking, or multitask label	Rare events, task structure, and heterogeneous label costs	Coverage of rare or high-risk cases and utility per unit cost

Table 9 complements the domain-level matrix by summarizing representative lines of study within the four-axis framework. It clarifies which aspects of trustworthiness are explicitly addressed across different areas of the literature and which remain only partially addressed.

**Table 9:** Representative study-level synthesis under the four-axis framework.

Study line	Domain/task	Acquisition logic	Supervision unit	Realism element	Main limitation for trustworthiness
Bayesian deep AL (Gal et al., 2017)	Image classification	Bayesian/dropout uncertainty	Instance label	Deep-model uncertainty estimation	Efficiency depends strongly on uncertainty quality and benchmark assumptions
Ensemble AL (Beluch et al., 2018)	Image classification	Model disagreement	Instance label	Epistemic uncertainty via ensembles	Improved uncertainty comes with additional computational cost
Core-set AL (Sener and Savarese, 2018)	Computer vision	Coverage/diversity	Instance label	Batch selection and representation geometry	Coverage does not guarantee task-specific informativeness
BADGE-style batch AL (Ash et al., 2019)	Deep classification	Gradient diversity and uncertainty	Instance label	Batch-mode acquisition	Relies on representation quality and may still ignore annotation effort
Medical segmentation AL (Boehringer et al., 2023; Gaillochet et al., 2023; Li et al., 2023; Saeed et al., 2024)	Medical imaging	Uncertainty, interaction, task prioritization	Mask, patch, slice, or superpixel	Expert annotation burden and dense labels	Query count alone can misrepresent true clinical effort
Object-detection AL (Garcia et al., 2023; Chen et al., 2023; Bonković et al., 2026)	Detection/localization	Uncertainty, semi-supervision, cost-awareness	Image, region, or box	Localization burden and object density	Human effort depends on object complexity, not only image count
NLP/document AL (Riesener et al., 2024; Wataya et al., 2025; Hu et al., 2025)	NER, reports, misinformation	Uncertainty and iterative selection	Token, span, sentence, or document	Semantic expertise and interpretive burden	Cognitive cost and annotator disagreement are often under-modeled
Systematic review AL (Ferdinands et al., 2023; Teijema et al., 2025)	Evidence screening	Prioritization for discovery/recall	Abstract or record judgment	Reviewer workload, stopping, recall	Results depend on prevalence, stopping rule, and classifier choice
Federated AL (Deng et al., 2025)	Medical classification	Querying under distributed training	Instance label	Privacy-sensitive data access	Acquisition must be coordinated under governance and communication constraints
Rarity-/shift-aware AL (Yang et al., 2025; Benhafid and Selouani, 2026)	Target shift/rare detection	Shift or rarity sensitivity	Task-dependent	Non-i.i.d. deployment and rare cases	Requires credible target-domain or rarity assumptions

### 4.1 Medical imaging

Medical imaging offers a strong motivation for AL because expert labels are expensive, dense annotations are time-consuming, and errors can have clinical consequences. In brain tumor segmentation and related medical imaging tasks, AL has been used to reduce manual delineation effort while maintaining strong segmentation performance (Boehringer et al., 2023). More recent studies go beyond simple uncertainty sampling by incorporating stochastic batching, interactive annotation, task prioritization, and alternative annotation units such as slices, patches, and superpixels (Gaillochet et al., 2023; Li et al., 2023; Saeed et al., 2024). Privacy-sensitive medical applications also motivate the combination of AL with federated learning, in which annotation efficiency and data governance must be addressed together (Deng et al., 2025).

The distinctive lesson from medical imaging is that supervision granularity is inseparable from clinical effort. A volumetric mask, a patch-level label, a slice annotation, and a superpixel correction represent different forms of labor and should not be treated as equivalent simply because each is counted as one query. This domain, therefore, exposes the weakness of label-count-only evaluation and shows why expert effort, adjudication, and workflow realism should be part of AL assessment.

Medical imaging also illustrates the limits of uncertainty-centric acquisition. Dense volumetric data often contain spatial redundancy, ambiguous boundaries, and nonuniform annotation burden. Pure uncertainty sampling may identify difficult regions, but it may also over-sample redundant or clinically marginal areas. The more persuasive medical imaging studies, therefore, tend to link acquisition to structured annotation workflows rather than to uncertainty scores alone.

### 4.2 Computer vision and object detection

Object detection poses a distinct supervision-allocation problem. Unlike image classification, detection requires localization, and uncertainty may arise from object presence, class identity, boundary placement, object density, or overlapping proposals. Review work in object-detection AL shows a diverse literature but also one marked by heterogeneous assumptions and evaluation protocols (Garcia et al., 2023). Recent work has shifted toward lightweight uncertainty estimation, semi-supervised acquisition, dynamic budgets, and cost-aware detection pipelines (Chen et al., 2023; Wang and Stavness, 2025; Bonković et al., 2026; Li et al., 2026).

The distinctive lesson from object detection is that annotation cost depends strongly on visual and spatial complexity. Two images may count equally in a benchmark yet require very different amounts of labeling effort due to object density, small objects, occlusion, boundary ambiguity, or localization difficulty. This makes detection especially sensitive to cost-aware and diversity-aware acquisition.

Object detection also underscores the importance of batch design. Since detection annotation is usually performed in batches, a method that does not control redundancy may waste substantial effort on visually similar images or overlapping regions. Rarity-aware detection further shows that aggregate accuracy is insufficient when rare classes or operationally important objects are underrepresented (Benhafid and Selouani, 2026).

### 4.3 Natural language processing and document intelligence

In NLP and document intelligence, the dominant annotation burden is often semantic rather than spatial. The difficulty lies in interpreting context, ambiguity, discourse function, domain-specific meaning, or factual relevance. AL has been used for named entity recognition in requirements engineering, medical report classification, and low-resource misinformation detection, where pre-trained language models and iterative querying can reduce manual labeling effort (Riesener et al., 2024; Wataya et al., 2025; Hu et al., 2025).

The distinctive lesson from text-based domains is that annotation cost is cognitive and interpretive. Token-, span-, sentence-, relation-, and document-level labels differ not only in length but also in the amount of context and expertise required. Uncertainty in such tasks may indicate useful ambiguity, but it may also reflect inconsistent annotation, sparse class signals, or distributional mismatch between corpora.

This domain broadens the concept of AL beyond image-centered label saving. It shows that AL can be understood as a strategy for rationing scarce interpretive labor. For this reason, trustworthy AL in NLP should report not only per-query performance but also the type of annotation requested, the required expertise, and the robustness of the selected examples across corpora or deployment settings.

### 4.4 Systematic review automation

Systematic review automation is one of the clearest human-in-the-loop AL settings because the operational goal is explicit: reduce screening workload while maintaining very high recall. Simulation studies show that AL can improve discovery efficiency and reduce manual screening burden, but they also reveal sensitivity to classifier choice, prevalence rate, stopping rule, and evaluation protocol (Ferdinands et al., 2023; Teijema et al., 2025).

The distinctive lesson from this domain is that workflow-grounded evaluation is not optional. Time to discovery, work saved at high recall, transparent stopping behavior, and reviewer burden are central outcomes, not secondary metrics. A screening system that appears efficient under one prevalence regime or stopping criterion may be much less reliable under another.

Systematic review automation, therefore, makes the value of a query unusually transparent. A queried abstract is useful only insofar as labeling it reduces reviewer workload without compromising the substantive recall requirements of the review. This domain thus provides a strong example of AL evaluated against operational goals rather than abstract benchmark curves alone.

### 4.5 Structured prediction, ranking, sensing, and anomaly detection

AL also extends beyond standard classification into entity matching, anomaly detection, ranking-based medical assessment, and multitask sensing (Han and Li, 2024; Nixon et al., 2024; Kadota et al., 2024; Arefeen and Ghasemzadeh, 2025). These areas are important because they show that the queried supervision may be a match judgment, an anomaly confirmation, a ranking comparison, or a multitask label rather than an ordinary class label.

The key lesson from these tasks is that acquisition is often inseparable from task structure. In ranking, relative comparisons may be more natural than absolute labels. In anomaly detection, the most valuable cases may be rare, ambiguous, or unlike the dominant data distribution. In multitask sensing, heterogeneous label costs may be inherent to the problem. Such settings expose the limitations of AL methods designed only for balanced, instance-level classification.

Together, these domains justify a broader view of AL as adaptive allocation of supervision under heterogeneous constraints. They also show why evaluation must consider rare-case coverage, utility per unit cost, and task-specific value, rather than relying solely on average performance over standard benchmark splits.

### 4.6 Cross-domain synthesis

The domain evidence supports four broader conclusions. First, the practical meaning of a query is domain-dependent: one label may correspond to a simple class decision, a dense spatial mask, a semantic span, a relevance judgment, or a rare-event confirmation. Second, domains with dense structure, high redundancy, or complex annotation interfaces most clearly reveal the limits of pure uncertainty sampling. Third, the strongest evidence for AL tends to come from studies that link acquisition to real-world workflows, cost models, stopping rules, or deployment constraints. Fourth, AL is now broad enough to be understood less as a family of classifier heuristics and more as a general framework for selective supervision under cost, ambiguity, risk, and operational constraints.

These findings reinforce the four-axis framework developed in this review. Acquisition logic explains why a candidate is selected, but domain evidence shows that this is only one part of the problem. Supervision granularity determines the amount of human effort required; operational regime determines the conditions under which querying occurs; and evaluation realism determines whether the reported gains are credible beyond a benchmark. Across domains, the central question is therefore not simply whether AL reduces the number of labels, but whether it allocates human supervision in a way that is efficient, robust, accountable, and aligned with the intended workflow.

## 5 Evaluation realism and methodological fragility

A critical review of AL must examine not only the methods themselves but also the quality of the evidence supporting claims of their effectiveness. A method may appear label-efficient under an idealized benchmark protocol yet be far less useful under realistic constraints on annotation, deployment, or workflow. For this reason, evaluation realism is not a secondary reporting issue; it is central to interpreting AL results.

The term *methodological fragility* is used here to describe how sensitive AL conclusions are to evaluation choices, including the initial labeled set, random seed, budget schedule, batch size, model architecture, calibration quality, annotation-cost model, class imbalance, and train–test distribution alignment. This section does not claim to provide a formal meta-analysis of the field. Rather, it synthesizes recurrent evaluation weaknesses reported across recent AL studies and translates them into concrete standards for more credible evaluation.

### 5.1 Overreliance on idealized protocols

A large portion of AL evaluation still relies on idealized pool-based protocols in which the unlabeled pool and test set are drawn from the same distribution, label costs are treated as homogeneous, oracle labels are assumed to be correct, and retraining is performed under controlled benchmark conditions. Such protocols are useful for isolating the behavior of acquisition functions, but they can also exaggerate practical value. In real applications, the unlabeled pool may be biased, redundant, noisy, or distributionally mismatched with deployment data. Annotation may require expert input, dispute resolution, or multiple rounds of correction. The cost of acquisition may depend on the structure of the selected example rather than on query count alone.

This matters because AL is often motivated by practical efficiency. If the evaluation protocol ignores the conditions that make annotation expensive, the reported efficiency gains may not reflect real savings. For instance, a method that reduces the number of images queried may not reduce expert workload if the selected images require dense masks, multiple object boxes, difficult clinical adjudication, or lengthy interpretive decisions. Similarly, a method that performs well on balanced i.i.d. benchmarks may fail in a deployment environment with rare classes, domain shift, or changing data distributions.

A more realistic evaluation should therefore specify the data-access regime, the labeling unit, the annotation-cost assumption, the retraining schedule, and the deployment scenario being approximated. Without this information, it is difficult to determine whether an AL gain reflects a robust property of the acquisition method or a favorable artifact of the benchmark design.

### 5.2 The persistence of label-count metrics

The most common evaluation curve in AL plots predictive performance against the number of queried labels. This representation is simple and useful, but it can be misleading when labels are not equally costly. In many modern applications, one query may correspond to an image-level class label, a bounding box, a segmentation mask, a token span, a relevance judgment, or a pairwise ranking decision. These supervision units differ substantially in time, expertise, cognitive load, and quality-control requirements.

A label-count metric implicitly treats all queries as equivalent. This assumption is reasonable only when the annotation unit is homogeneous, and the effort per query is roughly constant. In dense prediction, object detection, document analysis, medical imaging, and systematic review screening, this assumption often fails. Therefore, cost-aware AL requires metrics that relate performance to annotation effort rather than to query count alone (Wang and Stavness, 2025; Arefeen and Ghasemzadeh, 2025; Bonković et al., 2026).

The appropriate cost metric may differ across domains. In segmentation, effort may be approximated by time, clicks, edited regions, or mask complexity. In object detection, it may depend on the number of objects, object size, occlusion, and localization difficulty. In NLP, cost may depend on span length, contextual ambiguity, required expertise, and annotator disagreement. In systematic review automation, cost may be measured by the screening workload saved at a fixed recall target. Thus, cost-aware evaluation should be explicit about what form of human effort is being measured or modeled.

### 5.3 Insufficient robustness testing

A second source of fragility concerns the stability of reported AL gains. Performance curves can vary substantially with the initial labeled set, random seed, budget schedule, class imbalance, and architecture. A method that outperforms a baseline in a single run may not do so consistently across different initializations or budget regimes. This issue is particularly important in low-label settings, where early acquisition decisions can strongly influence the learner's subsequent trajectory.

Robustness also encompasses sensitivity to label noise, calibration error, outliers, and distribution shift. Because many acquisition functions depend on uncertainty or model confidence, poor calibration can directly distort the query ranking. Similarly, uncertainty-based methods may over-query outliers, noisy samples, or examples far from the deployment distribution. Diversity- and representativeness-based methods may also fail if the representation space does not capture task-relevant structure.

For these reasons, a credible AL evaluation should report performance across multiple random seeds, multiple initial labeled sets, and several budget levels. It should also test whether conclusions hold under class imbalance, label noise, distribution shift, or changes in model architecture. Reporting only a single acquisition curve can obscure substantial variance and make fragile methods appear more reliable than they are.

### 5.4 Underreported workflow assumptions

Many AL studies describe acquisition functions in detail but provide limited information about the human workflow they are meant to support. Important questions are often left implicit. Who provides the labels? Are annotators experts or crowd workers? Is there a disagreement resolution? Are labels reviewed or adjudicated? How long does annotation take? Does the annotation interface influence cost? Are queried samples labeled independently, or are they presented in batches? Is retraining performed after every query, after each batch, or according to a fixed schedule?

These questions are not merely implementation details. They affect the practical meaning of AL. A method designed for one-at-a-time querying may not translate to a batch annotation platform. A method that assumes instantaneous oracle feedback may not fit clinical or industrial workflows where expert time is scheduled in sessions. A method that ignores annotator disagreement may overstate the reliability of the labels it acquires.

Workflow-grounded evaluation should therefore report the annotation setting as part of the experimental design. Even when real annotators are not used, a simulation should specify which aspects of the human process are idealized and which are modeled. This transparency allows readers to judge whether the reported gains are likely to transfer to real annotation workflows.

### 5.5 Comparability problems across tasks

A further difficulty is that AL results are often hard to compare across domains. A one-point improvement in classification accuracy, a Dice-score gain in segmentation, an increase in mean average precision for detection, a reduction in screening burden in systematic review, and improved rare-event recall in anomaly detection are not directly comparable. The evaluation metric reflects the structure and risk profile of the task.

This heterogeneity is unavoidable, but it should be made explicit. Cross-domain comparison should focus less on whether one acquisition function universally dominates another and more on whether a method is evaluated with metrics appropriate to its task, annotation burden, and deployment goal. A clinically meaningful improvement in segmentation may justify substantial expert effort, whereas a small gain in a low-risk classification task may not. Similarly, a rare-event detection system may be judged by rare-class coverage rather than by average accuracy.

The implication is that AL benchmarks should be interpreted within their domain context. Universal rankings of acquisition strategies are often less informative than conditional statements about when a strategy works, under what cost model, and for which supervision unit. This is why evaluation realism is included as a core axis of the taxonomy rather than treated as a purely external reporting concern.

### 5.6 From evaluation weaknesses to reporting standards

The evaluation problems discussed above can be translated into practical reporting standards. Table 10 summarizes the main weaknesses and the corresponding requirements for a more credible AL evaluation. The purpose is not to impose a single benchmark protocol on all domains, but to make the evidential basis of AL claims more transparent and comparable.

**Table 10:** From evaluation weakness to actionable reporting standard.

Evaluation weakness	Recommended reporting standard	Reason
Single-seed or single-split results	Report mean and dispersion over multiple seeds, initial labeled sets, and train–validation splits.	Distinguishes robust acquisition gains from favorable initialization effects.
One arbitrary budget point	Evaluate across multiple labeling budgets and show complete acquisition curves.	Prevents conclusions that hold only at a selected budget.
Weak or incomplete baselines	Include random sampling, uncertainty sampling, diversity-aware baselines, and task-relevant cost-aware baselines when appropriate.	Ensures that proposed methods outperform simple and competitive alternatives.
Label-count-only evaluation	Report performance against annotation effort, time, clicks, mask complexity, number of boxes, or another justified cost proxy.	Aligns evaluation with the real motivation of AL: reducing human supervision burden.
No robustness analysis	Test sensitivity to class imbalance, label noise, calibration error, data redundancy, and distribution shift.	Assesses whether gains remain meaningful under realistic perturbations.
Unspecified annotation workflow	Describe annotator expertise, interface, batch size, adjudication, disagreement resolution, and quality-control procedures.	Clarifies what human process the AL system is intended to support.
Unreported computational overhead	Report acquisition-time cost, retraining frequency, memory requirements, and inference overhead.	Determines whether the acquisition itself introduces impractical cost.
Domain-inappropriate metrics	Use evaluation metrics aligned with the task, supervision unit, and operational risk.	Avoids misleading cross-domain comparisons and supports deployment-relevant interpretation.

### 5.7 Synthesis

Evaluation realism changes how AL should be interpreted. Under idealized conditions, an acquisition function may appear to improve accuracy per label. Under realistic conditions, the same method must also justify the effort it requires, the assumptions it makes about the annotation process, the stability of its gains, and its suitability for deployment. This is why AL evaluation should be multidimensional: it should consider predictive performance, annotation effort, robustness, workflow compatibility, computational overhead, and domain-specific risk.

The broader conclusion is that methodological fragility is not merely a weakness of individual studies; it reflects a structural tension in AL evaluation. The field seeks generalizable acquisition principles, yet the value of a query is often task-, cost-, and workflow-specific. A trustworthy AL system should therefore be evaluated not only as a model-selection mechanism but also as a human-machine decision process for allocating scarce supervision. This perspective directly connects the evaluation critique to the design principles and research agenda developed in the following sections.

## 6 Design principles for trustworthy active learning

The preceding sections have argued that AL is no longer adequately understood as a narrow problem of identifying the most uncertain samples. When AL is examined across acquisition families, supervision granularities, operational regimes, application domains, and evaluation protocols, a broader picture emerges. The modern field is heterogeneous but not formless. Across this diversity, several design principles can be distilled. These principles are not rigid rules, nor do they eliminate the need for task-specific judgment. Rather, they identify the recurring conditions under which AL is more likely to be trustworthy, practically meaningful, and methodologically credible.

The principles below are organized around three transitions. First, AL should move from uncertainty-only selection toward multi-criteria acquisition. Second, it should shift from label-count reduction to explicit modeling of the cost of human supervision. Third, it should move from idealized benchmark gains toward trustworthy evidence grounded in robustness and workflow realism.

### 6.1 From uncertainty to multi-criteria acquisition

One of the clearest conclusions of the modern literature is that raw uncertainty is too fragile to serve as the sole basis of acquisition in many realistic settings. Calibration error, redundancy, outliers, ambiguous boundary cases, and distributional shift can all distort uncertainty estimates. A method that relies entirely on uncertainty may confuse model ignorance with data pathology, or repeatedly spend its annotation budget on highly similar, ambiguous examples.

This does not imply that uncertainty should be abandoned. On the contrary, uncertainty remains one of the most valuable signals available to an active learner. The design lesson is more precise: uncertainty should usually be embedded within a broader acquisition logic. In practical systems, it is most effective when combined with structural correctives such as diversity, representativeness, cost awareness, or shift awareness. The goal is therefore not to replace uncertainty, but to prevent it from becoming a one-dimensional theory of acquisition value.

Batch composition is central to this multi-criteria view. In practice, AL is rarely performed on a single sample at a time. Human annotators often work in batches, and retraining after every individual label is frequently infeasible. Under such conditions, redundancy control becomes a structural requirement rather than a cosmetic refinement. A batch of individually informative but mutually redundant samples is an inefficient use of annotation effort.

Diversity is therefore best understood not as an optional supplement to informativeness, but as a condition under which informativeness becomes operationally useful. Under batch querying, the value of a candidate sample depends partly on what else has already been selected. Strong acquisition design should therefore consider not only each sample's score in isolation but also the collective composition of the queried batch.

### 6.2 From label counts to human supervision cost

A second principle follows directly from the practical motivation behind AL. If AL is intended to reduce real human effort, the relevant objective cannot be performance per label alone. It must be performance per unit of effort. That effort may involve time, clicks, spatial delineation burden, cognitive load, adjudication complexity, disagreement resolution, or the need for scarce domain expertise.

This point is especially important in domains such as segmentation, object detection, systematic review screening, and multitask sensing, where query cost is highly nonuniform. A method that reduces the number of queried examples may still fail to reduce the real workload if the selected examples require dense masks, difficult bounding boxes, expert interpretation, or repeated adjudication. Conversely, a method based on weak, partial, or interactive supervision may produce smaller per-query gains but greater savings in total human effort.

For this reason, annotation cost should be treated as a first-class quantity in trustworthy AL. It should not remain implicit in the background of the problem. A method that saves labels but increases real annotation burden should not be counted as efficient in any substantive sense. Cost-aware evaluation should therefore specify what type of human effort is being measured or modeled and why that cost proxy is appropriate for the task.

Closely related to cost is the granularity of supervision. The choice of what to query is not merely a property of the dataset; it is part of method design. A system that queries full images, one that queries superpixels, one that requests token spans, and one that asks for pairwise judgments are not simply variants of the same labeling process. They make different commitments about which form of supervision is worth purchasing and how to attack the annotation bottleneck.

The practical success of AL often depends less on the abstract acquisition score than on whether the form of queried supervision matches the true burden of the workflow. Strong AL systems are therefore not designed around a generic notion of sample selection. They are designed around the actual annotation bottleneck. In this sense, supervision granularity belongs to the core design space of AL rather than to its descriptive background.

### 6.3 From benchmark gains to trustworthy evidence

A third principle concerns evidential standards. The literature increasingly shows that many AL gains are more fragile than idealized benchmark results suggest. Performance can vary with model architecture, random seed, initial labeled set, budget schedule, class imbalance, calibration quality, label noise, and distributional shift. Under these conditions, robustness cannot be inferred from success on a single benchmark configuration.

Trustworthy AL, therefore, requires that empirical claims be tested rather than merely asserted. Methods should be evaluated across multiple seeds, initial labeled sets, budget levels, and realistic perturbations whenever possible. This is not simply a matter of statistical thoroughness. It is part of the substantive meaning of trustworthiness. An AL method that works only under narrow and favorable conditions may still be scientifically interesting, but it should not be presented as a generally reliable solution.

Workflow-grounded evaluation provides the strongest form of applied evidence. The most convincing AL studies are not necessarily those with the most sophisticated acquisition functions, but those that link performance directly to human processes and measurable workload savings. Domains such as systematic review screening and structured medical annotation are especially valuable in this respect because they require AL to be evaluated in terms of operationally meaningful outcomes rather than abstract benchmark curves alone.

The gold standard for applied AL is therefore not simply higher performance at a lower label count, but a demonstrable reduction in real human effort under the actual conditions of the task. When methods are evaluated in this way, the practical meaning of the acquisition strategy becomes clearer. Workflow-grounded evaluation provides the strongest bridge between methodological innovation and trustworthy deployment.

### 6.4 Synthesis

Taken together, these principles point toward a more mature conception of AL. Trustworthy AL is not defined by any single acquisition score or by a narrow contest among query heuristics. It is defined by the quality of the supervision-allocation system as a whole: whether uncertainty is properly contextualized, whether annotation cost is made explicit, whether the form of queried supervision matches the real bottleneck, whether batch redundancy is controlled, whether empirical claims are robust, and whether evaluation remains tied to actual human work.

These principles also clarify the relationship between the four-axis taxonomy and the practical design of AL systems. Acquisition logic explains why a sample is selected; supervision granularity specifies the type of human effort required; operational regime determines how querying is integrated into a learning workflow; and evaluation realism determines whether the resulting claims are credible. A trustworthy AL pipeline should align all four dimensions rather than optimizing one in isolation.

The principles do not close the field. On the contrary, they open it to a more realistic and more ambitious agenda. They suggest that the future of AL lies not in ever more refined one-dimensional scoring rules but in better integration of machine learning, human factors, cost modeling, robustness analysis, and deployment-sensitive evaluation. This transition motivates the research agenda developed in the following section.

## 7 Open problems and a research agenda

The current fragmentation of the field should not be read solely as a weakness. It also reveals where the next generation of research questions is emerging. The research agenda is therefore organized around concrete methodological questions, each linked to the same underlying problem: how to coordinate machine inference, human labor, and deployment-sensitive judgment under constrained supervision budgets. Table 11 summarizes the main open problems and methodological directions that define the proposed research agenda for trustworthy active learning.

**Table 11:** Actionable research agenda for trustworthy active learning.

Open problem	Current limitation	Concrete research question	Possible methodological direction	Evaluation requirement
Foundation models	Pre-training changes uncertainty and reduces the meaning of classical sample selection	Which examples, prompts, tasks, or corrections should be actively supervised?	Query selection over samples, prompts, rationales, corrections, and domains	Test across prompt variants, target domains, and calibration conditions
Stopping criteria	Many studies fix arbitrary budgets rather than deciding when to stop	When does another query no longer justify cost, delay, or risk?	Cost-sensitive stopping rules using expected value of information and task risk	Report recall, residual uncertainty, saved effort, and failure cases
Cost modeling	Query count is still used as a proxy for effort	How can time, clicks, expertise, fatigue, adjudication, and correction effort be combined?	Multidimensional cost models linked to annotation interfaces	Validate cost proxies against observed annotation time or expert workload
Fairness and safety	Average label efficiency may neglect rare or high-risk groups	How should AL protect underrepresented, rare, or safety-critical cases?	Risk-aware, group-aware, and rarity-aware acquisition objectives	Report subgroup coverage, rare-class recall, and harm-sensitive metrics
Benchmarking realism	Rankings vary with seeds, budgets, datasets, and protocols	Which benchmark conditions are necessary for credible comparison?	Shared protocols for seeds, budgets, baselines, cost, shift, and reporting	Require multi-seed, multi-budget, cost-aware, and robustness-oriented comparisons

The subsections that follow expand on these agenda items and clarify that they are not isolated technical problems. They are interconnected aspects of the same broader challenge: designing active-learning systems that allocate human supervision under realistic constraints while remaining reliable, accountable, and useful in deployment.

### 7.1 Foundation models and cross-task transfer

Large pre-trained and multimodal models are reshaping the economics of AL in fundamental ways. In some settings, strong pre-training may reduce the marginal value of iterative querying by making models more label-efficient from the outset. In other settings, however, foundation models introduce new complications rather than eliminating the need for AL. These include prompt sensitivity, unstable uncertainty estimates, interactions between in-context learning and acquisition, and new forms of cross-task transfer that make it less clear what a “useful” query should look like (Zhang and Takada, 2025).

This is therefore not merely a matter of applying classical AL methods to larger models. The rise of foundation models changes the underlying problem formulation. If pre-training, adaptation, and prompting already redistribute the need for supervision, then the role of AL must be reconsidered at the level of system design. One of the major open questions is whether AL in this setting should still focus on sample selection in the classical sense, or instead allocate supervision across examples, prompts, rationales, tasks, domains, and correction mechanisms.

This also creates new evaluation requirements. Foundation-model AL should be tested not only on a single dataset or prompt format but also across prompt variants, target domains, calibration conditions, and adaptation regimes. Otherwise, it will remain unclear whether active supervision improves the underlying task, the prompt interface, the adaptation process, or merely a narrow experimental configuration.

### 7.2 Stopping criteria

A second open problem is operational yet fundamental: when should querying stop? In many workflows, the central decision is not only which item to label next but also when the expected value of another label no longer justifies its cost. This problem remains underdeveloped in much of the literature, despite being one of the most practically important aspects of deployment.

The difficulty is that stopping cannot be reduced to a purely statistical threshold. In real systems, it depends on cost, task risk, desired recall, domain-specific tolerance for error, and the availability of additional annotation resources. A stopping rule acceptable in exploratory document screening may be unacceptable in a safety-critical medical workflow. The research challenge is therefore to develop stopping criteria that are not only mathematically principled but also operationally interpretable and sensitive to the consequences of under- or over-querying.

A trustworthy stopping criterion should therefore combine statistical evidence with cost- and risk-sensitive reasoning. It should ask whether another query is expected to improve performance, reduce uncertainty, increase coverage of high-risk cases, or prevent a consequential failure sufficiently to justify the additional human effort. This shifts the decision from an arbitrary budget allocation to a supervision-allocation decision.

### 7.3 Multidimensional cost modeling

The field still lacks a sufficiently rich theory of annotation cost. Time, clicks, cognitive effort, fatigue, interface complexity, training requirements, and adjudication demands can all matter, yet most AL formulations still treat cost in a simplified or implicit manner. This leaves a major gap between what AL claims to optimize and what many applications actually require.

A more mature approach to cost would treat the annotation burden as multidimensional rather than scalar. It would also recognize that cost interacts with supervision granularity, expertise requirements, interface design, disagreement resolution, and downstream correction. This is an area where AL would benefit from deeper integration with human-computer interaction, labor modeling, and work measurement. Without such progress, the field risks continuing to optimize convenient proxies for effort rather than effort itself. Future work should therefore validate cost models against observed annotation behavior. A credible cost proxy should be compared with measures such as annotation time, number of clicks, correction burden, expert fatigue, disagreement frequency, or other measurable indicators of effort. Without this validation, cost-aware acquisition may become only a formal extension of AL rather than a genuinely more realistic framework.

### 7.4 Fairness, coverage, and safety

Another open frontier concerns fairness and coverage. AL methods are not neutral with respect to representation. By directing limited annotation resources toward some cases rather than others, they can either amplify or mitigate imbalance in the resulting labeled dataset. This raises important questions about how querying policies affect underrepresented groups, rare but high-risk cases, and decision regions where errors are especially costly. This challenge is particularly acute in safety-critical domains, where the most practically important cases may also be the least common. A naïve acquisition strategy may improve average efficiency while neglecting precisely those cases that matter most from an ethical or safety perspective. The research problem here is not only to make AL more efficient but also to make its notion of value more responsive to asymmetries in harm, risk, and representational coverage.

Future AL methods should therefore incorporate coverage and harm-sensitive objectives more explicitly. This does not mean that every AL system must use the same fairness metric. Rather, it means that the relevant subgroup structure, rare-case risk, and safety-critical failure modes should be clearly stated and evaluated. In high-stakes settings, a method that improves average label efficiency while reducing attention to rare or vulnerable cases cannot be considered trustworthy.

### 7.5 Benchmarking for realism

The reproducibility and comparability of AL research will improve only when the field adopts stronger, shared standards for realism. At present, too much work still relies on narrow protocols that obscure variance, ignore the heterogeneous annotation burden, or fail to test robustness under realistic perturbations. Recent critiques have made this increasingly difficult to ignore (Mittal et al., 2025). What is needed is not simply more benchmarks, but better benchmark design. Realistic benchmarking should include multi-seed reporting, cost-aware metrics, robustness analyses, clearer workflow assumptions, and explicit treatment of distribution shift where relevant. In other words, the field needs shared standards for realism, not only shared datasets. Without this, methodological comparison will remain weaker than the ambition of the literature requires.

Benchmarking realism should also include a strong baseline discipline. New acquisition methods should be compared not only with random sampling but also with competitive baselines for uncertainty, diversity, batch-mode, and cost-awareness, when relevant. They should also report computational overhead, because an acquisition strategy that saves labels but imposes prohibitive training or inference cost may not be operationally useful.

### 7.6 Toward a broader theory of selective supervision

The open problems above point toward a broader conceptual challenge. AL has outgrown the simple picture of a model that requests labels for uncertain points. The literature reviewed in this article suggests that AL is increasingly a problem in designing adaptive supervision systems under practical constraints. Such systems must decide what form of supervision to request, from whom, at what cost, under what operational regime, and with what evidential standard for claiming success.

Foundation models, stopping criteria, richer cost modeling, fairness-sensitive querying, and realistic benchmarking are therefore not disconnected future directions. They all arise from the same transformation: AL is becoming a problem of coordinating machine inference, human labor, and deployment-sensitive judgment under limited resources. This is why the agenda should not be interpreted as a loose collection of topics but as a set of interconnected methodological challenges arising from the supervision-allocation view.

Future progress will likely depend on integrating machine learning, human factors, and operational decision theory more tightly than has been common so far. This does not mean abandoning algorithmic innovation. It means situating algorithmic innovation inside a broader framework that takes the realities of human work, deployment constraints, heterogeneous annotation value, and trustworthiness requirements seriously. Such a framework would strengthen the design of AL methods and clarify what the field is ultimately trying to optimize. The future of AL will therefore be shaped less by isolated improvements in acquisition heuristics than by the effort to build a more general theory of selective supervision. The fragmentation of the field should be interpreted with caution: it is a source of difficulty, but it is also a sign that the scope of the problem has expanded. The next stage of research will depend on whether this expansion can be turned into a coherent, realistic program for the trustworthy allocation of supervision.

## 8 Conclusion

This study provided a critical review and synthesis of modern AL. Its central argument is that AL should no longer be understood primarily as a collection of uncertainty-based heuristics, but as a broader set of methods for allocating costly human supervision under practical constraints. To organize this increasingly heterogeneous field, the review proposed a four-axis taxonomy based on acquisition logic, supervision granularity, operational regime, and evaluation realism. This framework helps explain why methods that appear similar at the level of query selection may differ substantially in the type of supervision they request, the workflows they assume, the costs they impose, and the strength of the evidence supporting their reported gains.

The synthesis developed throughout the review shows that AL can yield meaningful efficiency gains across domains, but that these gains are conditional rather than automatic. They depend on the quality of uncertainty estimation, the control of redundancy in batch selection, the realism of cost modeling, the robustness of acquisition under imbalance or distribution shift, and the suitability of the evaluation protocol. For this reason, future progress in AL is unlikely to come from one-dimensional acquisition scores alone. It will require treating AL as a multi-objective supervision-allocation problem, in which informativeness, coverage, cost, robustness, fairness, and workflow compatibility must be considered together.

The main challenge of AL has therefore shifted from identifying the most informative unlabeled sample to designing supervision-allocation pipelines that remain reliable under realistic annotation and deployment conditions. Whether AL becomes a stable component of trustworthy, data-efficient AI will depend on how seriously the field addresses this broader challenge: not only by proposing stronger acquisition functions but also by demonstrating that AL systems remain reliable, fair, transparent, cost-aware, robust, and accountable in the environments where they are actually used.
